# Supplementary material for: The Evolution and Characterization of the RNA Interference Pathways in Lophotrochozoa
Source: Genome Biol Evol. 2024 May 7;16(5):evae098. doi: 10.1093/gbe/evae098 (PMC11114477; doi:10.1093/gbe/evae098)
Supplement: evae098_Supplementary_Data [file evae098_supplementary_data.zip › Supplementary_materials.zip!/selected_miRNAs/NoGe_selected_miRNAs/mirdeep_pdf_files/NMRB01000320.1_Notospermus_geniculatus_strain_Ushimado_scaffold320,_whole_genome_shotgun_sequence_10298.pdf]

Provisional ID	:	NMRB01000320.1_Notospermus_geniculatus_strain_Ushimado_scaffold320,_whole_genome_shotgun_sequence_10298
Score total	:	37685.4
Score for star read(s)	:	3.9
Score for read counts	:	37676.7
Score for mfe	:	1.7
Score for randfold	:	0
Score for cons. seed	:	3
Total read count	:	73913
Mature read count	:	73389
Loop read count	:	0
Star read count	:	524

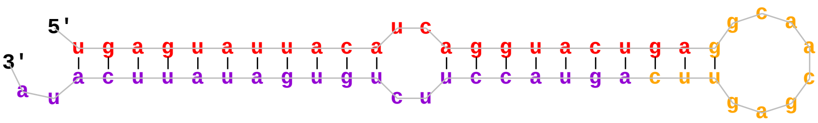

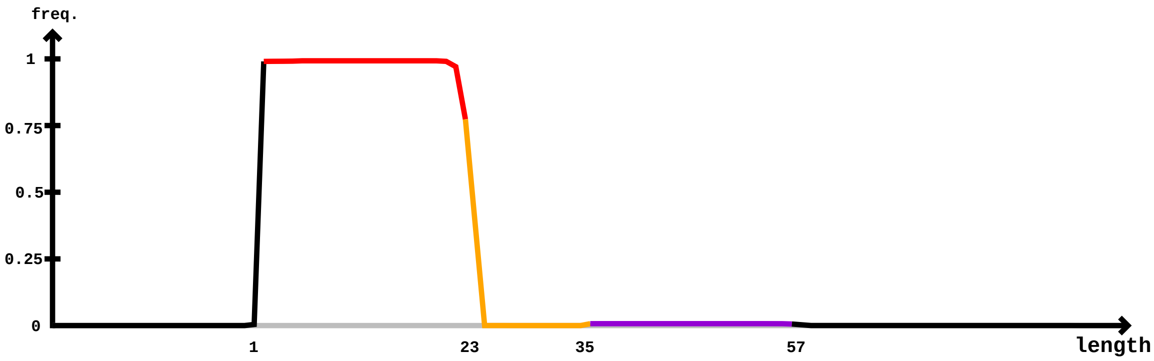


```
                         Mature                        Star
5'- aaaaguagacuucucaguuuugaguauuacaucagguacugaggcaacgaguucaguaccuucugugauauucauaacuuggaaguuguagacagcaggaagaccaacau  -3'   obs
    aaaaguagacuucucaguuuugaguauuacaucagguacugaggcaacgaguucaguaccuucugugauauucauaacuuggaaguuguagacagcaggaagaccaacau        exp
    .......(((((((.((((.(((((((((((..((((((((((........))))))))))..))))))))))).)))).)))))))..........((....)).....   reads   mm   sample
    ..................uugaguauuacaucagguacu.......................................................................   5       0    Fm2
    ..................uugaguauuacaucaggAacug......................................................................   1       1    Fm2
    ..................uugaguauuacaucagguacug......................................................................   20      0    Fm2
    ..................uugaguauuacaucaggGacug......................................................................   1       1    Fm2
    ..................Cugaguauuacaucagguacuga.....................................................................   2       1    Fm2
    ....................ugaguauuacaucagguac.......................................................................   5       0    Fm2
    ....................ugaguauuacaucagguacu......................................................................   148     0    Fm2
    ....................ugaguauuacaucagguacG......................................................................   1       1    Fm2
    ....................ugaguauuacaucagguGcu......................................................................   1       1    Fm2
    ....................ugaguauuacaucagguCcu......................................................................   1       1    Fm2
    ....................ugGguauuacaucagguacug.....................................................................   2       1    Fm2
    ....................ugaguauuacaucagguacGg.....................................................................   10      1    Fm2
    ....................ugaguauuacaucaggCacug.....................................................................   5       1    Fm2
    ....................ugaguauuacCucagguacug.....................................................................   4       1    Fm2
    ....................ugaguauuacaucaggGacug.....................................................................   8       1    Fm2
    ....................ugaguauuacaucagguCcug.....................................................................   9       1    Fm2
    ....................ugCguauuacaucagguacug.....................................................................   1       1    Fm2
    ....................ugaguauuacaucaUguacug.....................................................................   2       1    Fm2
    ....................Cgaguauuacaucagguacug.....................................................................   2       1    Fm2
    ....................ugaguauuacaucagguGcug.....................................................................   18      1    Fm2
    ....................ugaguauuacUucagguacug.....................................................................   1       1    Fm2
    ....................ugagCauuacaucagguacug.....................................................................   3       1    Fm2
    ....................ugaCuauuacaucagguacug.....................................................................   1       1    Fm2
    ....................ugaguauuacaucagguacuU.....................................................................   1       1    Fm2
    ....................ugaguauuacaucaggAacug.....................................................................   7       1    Fm2
    ....................ugaguauuacaucagguUcug.....................................................................   8       1    Fm2
    ....................ugaguauuacGucagguacug.....................................................................   2       1    Fm2
    ....................ugaguauuacaucagguacuA.....................................................................   6       1    Fm2
    ....................ugaguauuUcaucagguacug.....................................................................   1       1    Fm2
    ....................ugaguauuacauUagguacug.....................................................................   1       1    Fm2
    ....................Ngaguauuacaucagguacug.....................................................................   4       1    Fm2
    ....................ugaguauuacaucagguacug.....................................................................   2777    0    Fm2
    ....................ugaguauuAaucagguacug......................................................................   1       1    Fm2
```

Mature Star

```
aaaaguagacuucucaguuuugaguauuacaucagguacugaggcaacgaguucaguaccuucugugauauucauaacuuggaaguuguagacagcaggaagaccaacau
....................ugaguauuacaucagCuacug.....................................................................    1     1    Fm2
....................ugaguauuacaucagguacugU....................................................................    2902  1    Fm2
....................ugaguauuacGucagguacuga....................................................................    1     1    Fm2
....................ugagCauuacaucagguacuga....................................................................    1     1    Fm2
....................ugaguauuacaucaCguacuga....................................................................    1     1    Fm2
....................ugaguauuacaucaggGacuga....................................................................    5     1    Fm2
....................ugaguauuacaucaggAacuga....................................................................    6     1    Fm2
....................ugaguauAacaucagguacuga....................................................................    1     1    Fm2
....................ugaguauuacaucagguacuUa....................................................................    1     1    Fm2
....................Ngaguauuacaucagguacuga....................................................................    2     1    Fm2
....................ugaguGuuacaucagguacuga....................................................................    1     1    Fm2
....................ugaguauuacaucaggCacuga....................................................................    2     1    Fm2
....................ugaguauuacaucagguacuga....................................................................    1372  0    Fm2
....................ugaguauuacaucagguGcuga....................................................................    4     1    Fm2
....................ugaguauuacaucagguaGuga....................................................................    1     1    Fm2
....................ugaguauuacaucagguCcuga....................................................................    8     1    Fm2
....................ugaUuauuacaucagguacuga....................................................................    1     1    Fm2
....................ugaguauuacCucagguacuga....................................................................    1     1    Fm2
....................ugUguauuacaucagguacuga....................................................................    1     1    Fm2
....................ugaguauuUcaucagguacuga....................................................................    1     1    Fm2
....................ugaguauuacaucagguacugG....................................................................    94    1    Fm2
....................ugaguauuacaucagguUcuga....................................................................    1     1    Fm2
....................ugaguauuacaucagguacugaA...................................................................    11    1    Fm2
....................ugaguauuacaucagguacugaU...................................................................    217   1    Fm2
....................ugaguauuacaucagguacugUg...................................................................    3     1    Fm2
.....................gaguauuacaucagguacug.....................................................................    1     0    Fm2
.....................gaguauuacaucagguacugU....................................................................    3     1    Fm2
.......................guauuacaucagguacugU....................................................................    2     1    Fm2
......................................................aguaccuucugugauauuca....................................    4     0    Fm2
......................................................aguaccuucugugauaGucau...................................    1     1    Fm2
......................................................aguaccuucugugauauucau...................................    20    0    Fm2
......................................................aguaccuucugugauauucauU..................................    9     1    Fm2
......................................................aguaccuucugugauauAcaua..................................    1     1    Fm2
......................................................aguaccuucugugauauucaua..................................    147   0    Fm2
......................................................aguaccuuAugugauauucaua..................................    1     1    Fm2
......................................................Uguaccuucugugauauucaua..................................    2     1    Fm2

..................uugaguauuacaucagguacu.......................................................................    8     0    Fm1
..................uugaguauuacaucagguacug......................................................................    23    0    Fm1
..................uugaguauuacaucaggAacug......................................................................    1     1    Fm1
..................uugaguauuacaucagguacugU.....................................................................    47    1    Fm1
....................ugaguauuacaucaggua........................................................................    2     0    Fm1
....................ugaguauuacaucagguU........................................................................    2     1    Fm1
....................ugaguauuacaucagguac.......................................................................    26    0    Fm1
....................ugaguauuacaucagguGc.......................................................................    2     1    Fm1
....................ugaguauuacaucagguacu......................................................................    435   0    Fm1
....................ugaguauuacaucagguGcu......................................................................    2     1    Fm1
....................ugaguauuacCucagguacu......................................................................    1     1    Fm1
....................ugaguNuuacaucagguacu......................................................................    1     1    Fm1
....................ugaguauuacaucagguacG......................................................................    7     1    Fm1
....................Ngaguauuacaucagguacu......................................................................    1     1    Fm1
....................uCaguauuacaucagguacu......................................................................    1     1    Fm1
....................ugaguauuacaucagguUcu......................................................................    2     1    Fm1
....................ugGguauuacaucagguacu......................................................................    1     1    Fm1
....................ugaguauuacaucagguacA......................................................................    1     1    Fm1
....................ugaguauuacaucagguacuU.....................................................................    1     1    Fm1
....................ugaguauuacUucagguacug.....................................................................    4     1    Fm1
....................ugaguauuacaucagguacuC.....................................................................    1     1    Fm1
....................ugaguauCacaucagguacug.....................................................................    1     1    Fm1
....................ugCguauuacaucagguacug.....................................................................    5     1    Fm1
....................ugaguauuacaucGgguacug.....................................................................    1     1    Fm1
....................ugaguauuacaucagguCcug.....................................................................    13    1    Fm1
....................Cgaguauuacaucagguacug.....................................................................    1     1    Fm1
....................ugaguUuuacaucagguacug.....................................................................    1     1    Fm1
....................ugaguauuacaucagguacGg.....................................................................    14    1    Fm1
....................ugaguauuacaucagguacuA.....................................................................    2     1    Fm1
....................ugaguauuacaucaggCacug.....................................................................    11    1    Fm1
....................ugNguauuacaucagguacug.....................................................................    1     1    Fm1
....................ugaguauuCcaucagguacug.....................................................................    4     1    Fm1
....................ugaguauuacaucagguGcug.....................................................................    12    1    Fm1
```

```
                    Mature                            Star
aaaaguagacuucucaguuuugaguauuacaucagguacugaggcaacgaguucaguaccuucugugauauucauaacuuggaaguuguagacagcaggaagaccaacau
....................ugagCauuacaucagguacug.....................................................................  3     1  Fm1
....................ugaguauuacaucaggGacug.....................................................................  6     1  Fm1
....................ugaguauuacaucagguUcug.....................................................................  5     1  Fm1
....................ugagGauuacaucagguacug.....................................................................  1     1  Fm1
....................ugaguauuacaucagguacCg.....................................................................  1     1  Fm1
....................ugaguNuuacaucagguacug.....................................................................  1     1  Fm1
....................ugaguGuuacaucagguacug.....................................................................  1     1  Fm1
....................uAaguauuacaucagguacug.....................................................................  1     1  Fm1
....................Ngaguauuacaucagguacug.....................................................................  3     1  Fm1
....................ugaguauuacCucagguacug.....................................................................  1     1  Fm1
....................ugaguauuUcaucagguacug.....................................................................  2     1  Fm1
....................ugaguauuacGucagguacug.....................................................................  4     1  Fm1
....................ugaguauuGcaucagguacug.....................................................................  1     1  Fm1
....................uUaguauuacaucagguacug.....................................................................  1     1  Fm1
....................ugaguauuacaucaggAacug.....................................................................  6     1  Fm1
....................ugaguauuacaucagguacug.....................................................................  2983  0  Fm1
....................ugaguauuacaucagguacugU....................................................................  6012  1  Fm1
....................ugaguauuacaucagguCcuga....................................................................  9     1  Fm1
....................ugaguauuacaucaggGacuga....................................................................  3     1  Fm1
....................ugaguauuacaucagguUcuga....................................................................  6     1  Fm1
....................ugaguauuacaucUgguacuga....................................................................  1     1  Fm1
....................ugaguauuacaucagguGcuga....................................................................  3     1  Fm1
....................ugaguauuacUucagguacuga....................................................................  1     1  Fm1
....................ugaguauuacaucagguacCga....................................................................  1     1  Fm1
....................ugaguauuaUaucagguacuga....................................................................  9     1  Fm1
....................ugaguauuacCucagguacuga....................................................................  2     1  Fm1
....................ugaguauuacaucaggAacuga....................................................................  3     1  Fm1
....................ugaguauuacauUagguacuga....................................................................  2     1  Fm1
....................ugaguauuacGucagguacuga....................................................................  4     1  Fm1
....................ugaguauuaAaucagguacuga....................................................................  1     1  Fm1
....................ugaguNuuacaucagguacuga....................................................................  1     1  Fm1
....................ugaguauuacaucagguacuga....................................................................  1818  0  Fm1
....................ugUguauuacaucagguacuga....................................................................  1     1  Fm1
....................ugaguauuacaucagguacugC....................................................................  7     1  Fm1
....................ugaguauuacaucaggCacuga....................................................................  6     1  Fm1
....................ugaguauuacaucagguacugG....................................................................  167   1  Fm1
....................ugaguauuacaucGgguacuga....................................................................  1     1  Fm1
....................ugaguauuacaucagguacugaU...................................................................  433   1  Fm1
....................ugaguauuacaucagguacugaA...................................................................  19    1  Fm1
....................ugaguauuacaucagguacugaC...................................................................  2     1  Fm1
....................ugaguauuacaucagguacugag...................................................................  3     0  Fm1
....................ugaguauuacaucagguacugaUg..................................................................  1     1  Fm1
.....................gaguauuacaucagguacu......................................................................  1     0  Fm1
.....................gaguauuacaGcagguacug.....................................................................  1     1  Fm1
.....................gaguauuacaucagguacugU....................................................................  1     1  Fm1
.......................guauuacaucagguacuga....................................................................  5     0  Fm1
........................uauuacaucagguacuga....................................................................  4     0  Fm1
......................................................aguaccuucugugauauuc.....................................  3     0  Fm1
......................................................aguaccuucugugauauuca....................................  3     0  Fm1
......................................................aguaccuucugugauauucau...................................  18    0  Fm1
......................................................aguaccuucuAugauauucau...................................  1     1  Fm1
......................................................aguaccuucugugauauucauU..................................  2     1  Fm1
......................................................aguaccuucugugauauucaua..................................  37    0  Fm1
............................................................................acuuggaaguuguagacagcaggaagacc.....  4     0  Fm1

..aaguagacuucuUaguuu..........................................................................................  1     1  Fm3
...................uugaguauuacaucagguacu......................................................................  3     0  Fm3
...................uugaguauuacaucagguacug.....................................................................  39    0  Fm3
...................uugaguauuacaucagguacugU....................................................................  128   1  Fm3
....................ugaguNuuacaucagguac.......................................................................  2     1  Fm3
....................Cgaguauuacaucagguac.......................................................................  1     1  Fm3
....................ugaguauuacaucagguCc.......................................................................  2     1  Fm3
....................ugaguauuacaucagguac.......................................................................  91    0  Fm3
....................ugaguauuacaucaggAac.......................................................................  1     1  Fm3
....................ugaguauuacaucaggGacu......................................................................  2     1  Fm3
....................ugaguauuacaucagguUcu......................................................................  1     1  Fm3
....................ugaguauuacUucagguacu......................................................................  1     1  Fm3
....................ugaguauuacaucagguacu......................................................................  469   0  Fm3
....................ugaguauuacaucagguCcu......................................................................  3     1  Fm3
....................ugaguNuuacaucagguacu......................................................................  1     1  Fm3
```

Mature Star

```
aaaaguagacuucucaguuuugaguauuacaucagguacugaggcaacgaguucaguaccuucugugauauucauaacuuggaaguuguagacagcaggaagaccaacau
....................ugGguauuacaucagguacu......................................................................  1      1  Fm3
....................ugaguauuacGucagguacu......................................................................  2      1  Fm3
....................ugaguauuacauUagguacu......................................................................  1      1  Fm3
....................ugagCauuacaucagguacug.....................................................................  1      1  Fm3
....................ugaguauuacaucagguGcug.....................................................................  25     1  Fm3
....................ugaguNuuacaucagguacug.....................................................................  1      1  Fm3
....................ugaguauuacaucGgguacug.....................................................................  1      1  Fm3
....................ugaguauuacaucagguacuA.....................................................................  2      1  Fm3
....................ugaguauuaUaucagguacug.....................................................................  1      1  Fm3
....................ugaguauuacaucagCuacug.....................................................................  3      1  Fm3
....................ugaguauuacaucaggCacug.....................................................................  14     1  Fm3
....................ugaguauuacaAcagguacug.....................................................................  1      1  Fm3
....................ugaguauuacaucaUguacug.....................................................................  4      1  Fm3
....................ugGguauuacaucagguacug.....................................................................  4      1  Fm3
....................ugaguauuaAaucagguacug.....................................................................  1      1  Fm3
....................ugaguauuacaucagguCcug.....................................................................  26     1  Fm3
....................ugaguaCuacaucagguacug.....................................................................  1      1  Fm3
....................ugaguCuuacaucagguacug.....................................................................  1      1  Fm3
....................uUaguauuacaucagguacug.....................................................................  1      1  Fm3
....................ugCguauuacaucagguacug.....................................................................  2      1  Fm3
....................ugaguauuacGucagguacug.....................................................................  9      1  Fm3
....................ugaguauCacaucagguacug.....................................................................  6      1  Fm3
....................ugaguauuacaucagAuacug.....................................................................  1      1  Fm3
....................ugaguauuacaucagguUcug.....................................................................  9      1  Fm3
....................uAaguauuacaucagguacug.....................................................................  5      1  Fm3
....................ugaguauuacauAagguacug.....................................................................  1      1  Fm3
....................ugaguauuacaucagguacuU.....................................................................  2      1  Fm3
....................ugaguauuacauUagguacug.....................................................................  2      1  Fm3
....................ugaguauuacaucagguaUug.....................................................................  8      1  Fm3
....................ugaguauuacaucaggGacug.....................................................................  12     1  Fm3
....................ugaguauuacaucagguacCg.....................................................................  7      1  Fm3
....................Ngaguauuacaucagguacug.....................................................................  3      1  Fm3
....................ugaguauuacaucagguacuC.....................................................................  1      1  Fm3
....................ugaguauuacCucagguacug.....................................................................  3      1  Fm3
....................ugaguaGuacaucagguacug.....................................................................  1      1  Fm3
....................Cgaguauuacaucagguacug.....................................................................  2      1  Fm3
....................ugaguauuacaucaggAacug.....................................................................  14     1  Fm3
....................ugaguauuacUucagguacug.....................................................................  3      1  Fm3
....................ugaguauuacaucagguacug.....................................................................  5863   0  Fm3
....................ugaAuauuacaucagguacug.....................................................................  1      1  Fm3
....................ugaguaCuacaucagguacuga....................................................................  3      1  Fm3
....................ugaguauuacaCcagguacuga....................................................................  3      1  Fm3
....................ugUguauuacaucagguacuga....................................................................  1      1  Fm3
....................ugaUuauuacaucagguacuga....................................................................  5      1  Fm3
....................ugaguauuacaucagguacugU....................................................................  21371  1  Fm3
....................ugaguauuacGucagguacuga....................................................................  15     1  Fm3
....................ugaguauuacaucaggCacuga....................................................................  26     1  Fm3
....................ugaguauuacauGagguacuga....................................................................  1      1  Fm3
....................ugaguauuacaucaUguacuga....................................................................  1      1  Fm3
....................ugaguauuGcaucagguacuga....................................................................  1      1  Fm3
....................uAaguauuacaucagguacuga....................................................................  7      1  Fm3
....................ugaguauuCcaucagguacuga....................................................................  1      1  Fm3
....................ugaguNuuacaucagguacuga....................................................................  1      1  Fm3
....................ugaguaGuacaucagguacuga....................................................................  1      1  Fm3
....................ugaguauuacaucCgguacuga....................................................................  1      1  Fm3
....................ugaguauuacaucagguUcuga....................................................................  17     1  Fm3
....................ugGguauuacaucagguacuga....................................................................  5      1  Fm3
....................ugaguauuacaucaggGacuga....................................................................  22     1  Fm3
....................ugCguauuacaucagguacuga....................................................................  2      1  Fm3
....................ugaguauuacaucagguacugC....................................................................  10     1  Fm3
....................uUaguauuacaucagguacuga....................................................................  5      1  Fm3
....................ugaguGuuacaucagguacuga....................................................................  2      1  Fm3
....................ugaguUuuacaucagguacuga....................................................................  3      1  Fm3
....................ugagCauuacaucagguacuga....................................................................  5      1  Fm3
....................Cgaguauuacaucagguacuga....................................................................  1      1  Fm3
....................ugaguauuacauAagguacuga....................................................................  1      1  Fm3
....................ugaguauuacaucagguaUuga....................................................................  1      1  Fm3
....................ugaguauuacauUagguacuga....................................................................  8      1  Fm3
....................ugaguauuacaAcagguacuga....................................................................  1      1  Fm3
....................ugaguauuUcaucagguacuga....................................................................  3      1  Fm3
```

```
                    Mature                            Star
aaaaguagacuucucaguuuugaguauuacaucagguacugaggcaacgaguucaguaccuucugugauauucauaacuuggaaguuguagacagcaggaagaccaacau
....................ugaguauuacaucagguacuga....................................................................   8093   0   Fm3
....................ugaguauuacaucagCuacuga....................................................................   2      1   Fm3
....................ugaguauuacaucGgguacuga....................................................................   1      1   Fm3
....................ugagAauuacaucagguacuga....................................................................   1      1   Fm3
....................uCaguauuacaucagguacuga....................................................................   1      1   Fm3
....................ugaguauuacaucagguacuAa....................................................................   3      1   Fm3
....................ugaguauuacaucagguacAga....................................................................   1      1   Fm3
....................ugaguauuacaucaggAacuga....................................................................   10     1   Fm3
....................ugaguauuacaucagguacugG....................................................................   1      1   Fm3
....................ugaguauuacCucagguacuga....................................................................   5      1   Fm3
....................Ngaguauuacaucagguacuga....................................................................   5      1   Fm3
....................ugaguauuacaucagguCcuga....................................................................   30     1   Fm3
....................ugaguauuacaucagguGcuga....................................................................   32     1   Fm3
....................ugaguauuacaucagAuacuga....................................................................   2      1   Fm3
....................ugaguauuaUaucagguacuga....................................................................   11     1   Fm3
....................ugaguauuacUucagguacuga....................................................................   2      1   Fm3
....................ugaguauuacaucagguacugaC...................................................................   3      1   Fm3
....................ugaguauuacaucagguacugaU...................................................................   1482   1   Fm3
....................ugaguauuacaucagguacugaA...................................................................   93     1   Fm3
....................ugaguauuacaucagguacugag...................................................................   1      0   Fm3
....................ugaguauuacaucagguacCgaggc.................................................................   1      1   Fm3
....................ugaguauuacaucagguacugaggca................................................................   2      0   Fm3
.....................gaguauuacaucagguacug.....................................................................   6      0   Fm3
.....................Uaguauuacaucagguacug.....................................................................   1      1   Fm3
.....................gaguauuacaucagguacugU....................................................................   5      1   Fm3
.....................gaguauuacaGcagguacuga....................................................................   1      1   Fm3
.....................gaguauuacaucagguacuga....................................................................   6      0   Fm3
.....................gaguauuacaucagguacugaU...................................................................   1      1   Fm3
......................aguauuacaucagguacuga....................................................................   10     0   Fm3
......................aguauuacauAagguacuga....................................................................   1      1   Fm3
......................Uguauuacaucagguacuga....................................................................   1      1   Fm3
......................aguauuacaucagguacAga....................................................................   1      1   Fm3
.......................guauuacaucagguacuga....................................................................   5      0   Fm3
.......................guauuacaucagguacugaU...................................................................   1      1   Fm3
........................uauuacaucagguacuga....................................................................   69     0   Fm3
........................uauuacaucagguacugU....................................................................   2      1   Fm3
........................uauuacaucagguacuCa....................................................................   1      1   Fm3
........................uauuacaucagguacugaU...................................................................   2      1   Fm3
......................................................aguaccuucugugauauuc.....................................   4      0   Fm3
......................................................aguaccuucugugauauucau...................................   15     0   Fm3
......................................................aguaccuucugugauauCcau...................................   1      1   Fm3
......................................................aguaccuucugugauauucaua..................................   53     0   Fm3
......................................................aguaccuucugugauauAcaua..................................   1      1   Fm3
......................................................aguaccuucugugauauucauU..................................   2      1   Fm3
......................................................aguaccuucugugauauucauaa.................................   1      0   Fm3
.......................................................guaccuucugugauauucaua..................................   3      0   Fm3
........................................................uaccuucugugauauucauaaA................................   2      1   Fm3

.aaaguagacuucucaguuu..........................................................................................   1      0   Mm1
....................ugaguauuacaucagguac.......................................................................   3      0   Mm1
....................ugaguauuacaucagguacu......................................................................   66     0   Mm1
....................ugaguauuacaucagguCcu......................................................................   1      1   Mm1
....................ugaguauuacaCcagguacu......................................................................   1      1   Mm1
....................ugaguauuacaucaggCacug.....................................................................   5      1   Mm1
....................ugagCauuacaucagguacug.....................................................................   1      1   Mm1
....................ugaguauuUcaucagguacug.....................................................................   1      1   Mm1
....................ugaguauuacaucaggGacug.....................................................................   8      1   Mm1
....................ugaguauCacaucagguacug.....................................................................   1      1   Mm1
....................ugaguauuacaucagguacGg.....................................................................   5      1   Mm1
....................uAaguauuacaucagguacug.....................................................................   2      1   Mm1
....................ugaguauuacGucagguacug.....................................................................   3      1   Mm1
....................ugaguauuacaucaggAacug.....................................................................   3      1   Mm1
....................ugaguauuacaucagguUcug.....................................................................   3      1   Mm1
....................ugaguauuacaucagguGcug.....................................................................   6      1   Mm1
....................ugaguauuacaucagguacug.....................................................................   1457   0   Mm1
....................ugaguauuacaucagguCcug.....................................................................   3      1   Mm1
....................ugaguauuacCucagguacug.....................................................................   1      1   Mm1
....................ugagGauuacaucagguacug.....................................................................   1      1   Mm1
....................ugaguauuacUucagguacuga....................................................................   1      1   Mm1
....................ugaguauuacaucagguacuga....................................................................   1227   0   Mm1
```

```
                    Mature                            Star
aaaaguagacuucucaguuuugaguauuacaucagguacugaggcaacgaguucaguaccuucugugauauucauaacuuggaaguuguagacagcaggaagaccaacau
....................ugaguauuacaucagguGcuga....................................................................    4     1    Mm1
....................ugaguauuacaucaggGacuga....................................................................    4     1    Mm1
....................ugaguauuacaucaggCacuga....................................................................    6     1    Mm1
....................ugaguauuacaucagguacuUa....................................................................    1     1    Mm1
....................ugaguauuacCucagguacuga....................................................................    1     1    Mm1
....................ugaguauuacGucagguacuga....................................................................    1     1    Mm1
....................ugaguauuacaucagguacugG....................................................................    10    1    Mm1
....................uUaguauuacaucagguacuga....................................................................    1     1    Mm1
....................ugUguauuacaucagguacuga....................................................................    1     1    Mm1
....................ugaguauuaAaucagguacuga....................................................................    1     1    Mm1
....................ugCguauuacaucagguacuga....................................................................    1     1    Mm1
....................ugaguauuacauGagguacuga....................................................................    1     1    Mm1
....................ugaguauuacaucUgguacuga....................................................................    1     1    Mm1
....................ugaguauuacaucagguUcuga....................................................................    3     1    Mm1
....................uCaguauuacaucagguacuga....................................................................    1     1    Mm1
....................ugaguauuacaucaggAacuga....................................................................    3     1    Mm1
....................ugaguauuacaucGgguacuga....................................................................    1     1    Mm1
....................ugaguauuacaucagguacugU....................................................................    4711  1    Mm1
....................ugaguauuacaucagguCcuga....................................................................    1     1    Mm1
....................ugaguauuacaucagguacugaU...................................................................    189   1    Mm1
....................ugaguauuacaucagguacugaA...................................................................    33    1    Mm1
.....................gaguauuacaucagguacug.....................................................................    4     0    Mm1
......................aguauuacaucagguacugU....................................................................    1     1    Mm1
.......................guauuacaucagguacuga....................................................................    13    0    Mm1
........................uauuacaucagguacugaU...................................................................    4     1    Mm1
......................................................aguaccuucugugauauucau...................................    2     0    Mm1
......................................................aguaccuucugugauauucaua..................................    32    0    Mm1
......................................................aguaccuucuUugauauucaua..................................    1     1    Mm1

...................uugaguauuacaucagguacug.....................................................................    5     0    Mm2
...................uugaguauuacaucagguacugU....................................................................    10    1    Mm2
....................ugaguauuacaucagguac.......................................................................    9     0    Mm2
....................ugaguauuacaucagguGcu......................................................................    2     1    Mm2
....................ugaguauuacaucaggAacu......................................................................    1     1    Mm2
....................ugaguauuacaucagguacu......................................................................    147   0    Mm2
....................ugaguaNuacaucagguacu......................................................................    1     1    Mm2
....................ugaguauuacaucaggGacu......................................................................    2     1    Mm2
....................ugaguauuacaucagguacG......................................................................    1     1    Mm2
....................ugaguauuacaucaggCacug.....................................................................    5     1    Mm2
....................ugaguaNuacaucagguacug.....................................................................    1     1    Mm2
....................ugaguauuacaucagguCcug.....................................................................    2     1    Mm2
....................ugCguauuacaucagguacug.....................................................................    1     1    Mm2
....................ugaguauuacaucagguacug.....................................................................    634   0    Mm2
....................ugaguauuacaucagguacGg.....................................................................    3     1    Mm2
....................ugaguauuacaucagguacuA.....................................................................    1     1    Mm2
....................ugaguauuacCucagguacug.....................................................................    1     1    Mm2
....................ugGguauuacaucagguacug.....................................................................    1     1    Mm2
....................ugaguauuCcaucagguacug.....................................................................    1     1    Mm2
....................ugaguauuacaucagguGcug.....................................................................    3     1    Mm2
....................ugaguauuacaucaggGacug.....................................................................    2     1    Mm2
....................ugaguauuacaucaggAacug.....................................................................    1     1    Mm2
....................uUaguauuacaucagguacug.....................................................................    1     1    Mm2
....................ugagCauuacaucagguacug.....................................................................    1     1    Mm2
....................Ngaguauuacaucagguacuga....................................................................    2     1    Mm2
....................ugaguauuacaucaggAacuga....................................................................    1     1    Mm2
....................ugaguauuacaucagguacugG....................................................................    31    1    Mm2
....................ugaguauuacaucagguUcuga....................................................................    1     1    Mm2
....................ugaguauuaUaucagguacuga....................................................................    1     1    Mm2
....................ugaguauuacaucagguacuAa....................................................................    1     1    Mm2
....................ugaUuauuacaucagguacuga....................................................................    2     1    Mm2
....................ugaguauuacaucagguaUuga....................................................................    3     1    Mm2
....................ugaguauuacaucaggGacuga....................................................................    4     1    Mm2
....................ugaguauuacauUagguacuga....................................................................    1     1    Mm2
....................ugaguauuacaucagguacugU....................................................................    1814  1    Mm2
....................ugaguauuacaucagguGcuga....................................................................    5     1    Mm2
....................ugagGauuacaucagguacuga....................................................................    1     1    Mm2
....................ugaguauuacaucagguacugC....................................................................    7     1    Mm2
....................ugaguauuacaucagguCcuga....................................................................    2     1    Mm2
....................ugaguauuacaucagAuacuga....................................................................    1     1    Mm2
....................ugaguauuacaucagguacuga....................................................................    831   0    Mm2
```

Mature **Star**

```
aaaaguagacuucucaguuuugaguauuacaucagguacugaggcaacgaguucaguaccuucugugauauucauaacuuggaaguuguagacagcaggaagaccaacau
....................ugaguauGacaucagguacuga....................................................................    2     1    Mm2
....................ugaguauuacUucagguacuga....................................................................    1     1    Mm2
....................ugaguauuacCucagguacuga....................................................................    1     1    Mm2
....................ugaguauuacaucagguacugag...................................................................    1     0    Mm2
....................ugaguauuacaucagguacugaA...................................................................    20    1    Mm2
....................ugaguauuacaucagguacugaU...................................................................    144   1    Mm2
.....................gaguauuacaucagguacug.....................................................................    1     0    Mm2
.....................gaguauuacaucagguacuga....................................................................    1     0    Mm2
......................aguauuacaucagguacuga....................................................................    1     0    Mm2
.......................guauuacaucagguacuga....................................................................    2     0    Mm2
........................uauuacaucagguacuga....................................................................    8     0    Mm2
..........................................ggcaacgaguucaguaccuucugugauauucaua..................................    2     0    Mm2
.....................................................caguaccuucugugauauucau...................................    1     0    Mm2
......................................................aguaccuucugugauauuc.....................................    1     0    Mm2
......................................................aguaccuucugugauauuca....................................    3     0    Mm2
......................................................aguaccuucugugauaAucau...................................    1     1    Mm2
......................................................aguaccuucugugauauucau...................................    11    0    Mm2
......................................................aguaccuucugugauGuucau...................................    1     1    Mm2
......................................................aguaccuucugugauauCcaua..................................    1     1    Mm2
......................................................aguaccuucugugauauucauU..................................    1     1    Mm2
......................................................aguaccuucugugauauucaua..................................    68    0    Mm2
......................................................aguaccuucugugauauGcaua..................................    1     1    Mm2
......................................................aguaccuucugugauaGucaua..................................    1     1    Mm2
......................................................aguaccuucugugauauAcaua..................................    1     1    Mm2
.......................................................guaccuucugugauauucaua..................................    2     0    Mm2
............................................................................acuuggaaguuguagacagcaggaagac......    1     0    Mm2

...................uugaguauuacaucagguacug.....................................................................    1     0    Mm3
...................uugaguauuacaucagguacugU....................................................................    12    1    Mm3
...................Cugaguauuacaucagguacuga....................................................................    1     1    Mm3
...................uugaguauuacaucagguacugG....................................................................    1     1    Mm3
....................ugaguauuacaucagguac.......................................................................    9     0    Mm3
....................ugaguauuacaucagguacu......................................................................    149   0    Mm3
....................ugGguauuacaucagguacu......................................................................    1     1    Mm3
....................ugaguauuacaucagguacG......................................................................    2     1    Mm3
....................ugaguauuacaucagguGcug.....................................................................    2     1    Mm3
....................ugaguauuacCucagguacug.....................................................................    1     1    Mm3
....................ugaguauuacauUagguacug.....................................................................    1     1    Mm3
....................ugaguauuacaucagguacug.....................................................................    301   0    Mm3
....................ugaguauuacaucaggGacug.....................................................................    2     1    Mm3
....................ugaguauuacaucaggCacug.....................................................................    2     1    Mm3
....................ugaguauuacaucaggCacuga....................................................................    2     1    Mm3
....................ugaguauuacaucaggGacuga....................................................................    2     1    Mm3
....................ugaguauuacaucagguCcuga....................................................................    2     1    Mm3
....................ugaguauuacaucagguUcuga....................................................................    1     1    Mm3
....................ugaguauuacaucagguacuga....................................................................    756   0    Mm3
....................ugaguauuacaucagguacugU....................................................................    2114  1    Mm3
....................ugaguauuacaucagguaAuga....................................................................    1     1    Mm3
....................ugaguauuacaucagguGcuga....................................................................    4     1    Mm3
....................ugaguauuacaucagguacugC....................................................................    5     1    Mm3
....................ugaguauuacGucagguacuga....................................................................    2     1    Mm3
....................ugaguauuaUaucagguacuga....................................................................    2     1    Mm3
....................uAaguauuacaucagguacuga....................................................................    2     1    Mm3
....................ugaguauuacaucaggAacuga....................................................................    1     1    Mm3
....................ugaguauuacaucagguacCga....................................................................    1     1    Mm3
....................Cgaguauuacaucagguacuga....................................................................    2     1    Mm3
....................ugaguauuacaucagguacugG....................................................................    16    1    Mm3
....................ugaguauuacaucagguacugag...................................................................    1     0    Mm3
....................ugaguauuacaucagguacugaU...................................................................    343   1    Mm3
....................ugaguauuacaucagguacugaA...................................................................    24    1    Mm3
.....................gaguauuacaucagguacuC.....................................................................    1     1    Mm3
.......................guauuacaucagguacuga....................................................................    1     0    Mm3
.......................guauuacaucagguacugaU...................................................................    1     1    Mm3
........................uauuacaucagguacuga....................................................................    2     0    Mm3
......................................................aguaccuucugugauauucau...................................    4     0    Mm3
......................................................aguaccuucuCugauauucaua..................................    1     1    Mm3
......................................................aguaccuucugugauauCcaua..................................    1     1    Mm3
......................................................aguaccuucugugauauucaua..................................    57    0    Mm3
......................................................aguaccuucugugauauucauU..................................    2     1    Mm3
```
